# Virtual non-contrast images of detector-based spectral computed tomography in dogs: a promising alternative to true non-contrast images in veterinary medicine

**DOI:** 10.3389/fvets.2023.1251535

**Published:** 2023-12-01

**Authors:** Philipp Lietz, Manon Brüntgens, Adriano Wang-Leandro, Holger Andreas Volk, Sebastian Meller, Kristina Merhof

**Affiliations:** Clinic for Small Animals, University of Veterinary Medicine Hannover, Hanover, Germany

**Keywords:** SDCT dual-layer spectral detector CT, virtual-non contrast (VNC), spectral based images, dual energy, contrast - enhanced CT

## Abstract

**Introduction:**

In veterinary medicine, abdominal computer tomographic (CT) examinations regularly require a minimum of two scans, with a native scan (true unenhanced, TUE) as a reference for the subsequent contrast-enhanced CT scan (CECT). Spectral detector CT (SDCT) offers the possibility to calculate virtual non-contrast (VNC) images from the post-contrast scan, but this has not yet been investigated in veterinary medicine. The purpose of this study was to assess the reliability of VNC images for abdominal organs in 44 dogs without abdominal pathologies by evaluating their quantitative and qualitative parameters compared to TUE images. We hypothesized that the subtraction of iodine is sufficient in the VNC series compared to the TUE series and that the image quality of the SDCT series is superior to conventional CT images.

**Methods:**

Corresponding attenuation values in the VNC and TUE series regarding the regions of interest (ROI) in different parenchymal organs and major vessels of the abdominal cavity were assessed by means of a two one-sided *t*-test (TOST) and Bland–Altman plots. Additionally, the signal-to-noise ratio (SNR) was calculated for each ROI in the different series. In the second step, two board-certified veterinary radiologists made a qualitative assessment of VNC images vs. TUE images in consensus by rating the iodine subtraction, image noise, and image quality of VNC images based on a specific 5-point Likert scale.

**Results:**

The difference in corresponding Hounsfield units (HUs) between TUE and VNC images was less than 10 HU in 78.67% of all ROIs. Regarding the limit of less than 10 HU, in the performed TOST, significant *p*-values of < 0.05 were reached for the liver, spleen, pancreas, and musculature, implying equivalence of both modalities. The quality of spectral base image (SBI) data was rated equivalent to calculated conventional images in the subjective assessment by reaching an average Likert scale score of 3.2 points.

**Discussion:**

VNC images calculated from SDCT data prove a valid alternative to conventional TUE images in the abdominal organs of canine patients without abdominal pathology. VNC offers the possibility to reduce time under general anesthesia and minimize radiation exposure. Future studies are needed to prove the application of this method in clinically diseased patients.

## 1 Introduction

The acquisition of pre- and post-contrast abdominal computer tomographic (CT) scans is a commonly used procedure in veterinary medicine and is the method of choice, for example, in tumor staging and the distinction of focal masses. In a variety of pathologies, we rely on the differences in imaging characteristics in pre- and post-contrast series, for example, the differentiation of mineralization from contrast medium (e.g., in suspected urolithiasis), and we draw conclusions about lesion type by its contrast uptake (e.g., abscess vs. neoplasia), although there is a great overlap in these characteristics (1–3). An important advantage of acquiring and comparing pre- and post-contrast CT images is the characterization of malignant vs. benign lesions using quantitative and qualitative CT parameters. In this way, the study by Burti et al. (4) was able to describe a significant difference in pre-contrast CT HUs measured in liver tissue affected by malignant focal lesions compared to benign focal lesions. Nevertheless, in some patients, we could relinquish the pre-contrast series, but unfortunately, it is often uncertain whether a TUE scan is necessary before reviewing the CT study. Even if the need becomes apparent after the initial CECT scan during the same examination, performing a subsequent TUE scan is typically postponed to the following day to avoid measuring any residual iodine in the patient’s body.

The use of an SDCT, as a special form of dual-energy CT (DECT), for CECT scans eliminates the need to make a prior decision about whether a TUE scan is required. This is because the dual-energy scan should provide the necessary information through virtual non-contrast image reconstructions for all patients.

In conventional helical CT, images are created from voxels with gray levels represented as Hounsfield units (HUs) based on the X-ray absorption properties of tissues and materials (5)—however, these HU values are not specific for the different tissues and materials. Certain materials such as iodine and bone absorb high-energy radiation similarly, but they show distinct absorption values for low-energy radiation due to differences in the binding energy of the k-shell electrons. DECTs analyze the incoming polychromatic X-ray beam based on high- and low-energy levels and use these distinct attenuation values to provide additional information to conventional CTs with the created spectral-based image (SBI) data (6, 7). While conventional CT image data are simultaneously generated in the same scan (8, 9) based on this principle, SDCT provides enhanced diagnostic capability in human medicine, for example:

- Material Differentiation: Spectral CT can distinguish between certain types of tissues and materials based on their unique energy absorption curves. This allows us to distinguish between different substances that have similar Hounsfield units in conventional CT scans (5, 10).
- Improved Image Quality: By using spectral data, image artifacts can be reduced and image quality can be enhanced by combining the image information of low- and high-energy X-ray spectra. A low-energy spectrum improves signal-to-noise ratio and improves the visibility of contrast enhancement, while a high-energy spectrum can reduce metal artifacts (11, 12).
- Quantitative Analysis: Spectral CT enables quantitative analysis of tissue characteristics, such as iodine concentration and calcium quantification. This quantitative information can be valuable for monitoring disease progression and treatment response (10, 13).
- Retained Radiation Dose: The SDCT technology offers these advantages without an increased radiation dose for the patient, due to the analysis of the data from the conventional X-ray beam at the detector level (14).

Additionally, SDCT provides the possibility of reconstructing virtual non-contrast images from post-contrast spectral CT data by identifying and replacing iodine pixels. Studies in human medicine have demonstrated good to excellent comparability of VNC with TUE images when using different comparative methods, including subjective comparative assessments of VNC with TUE images by trained radiologists and objective analyses of SDCT quality parameters such as signal-to-noise ratio (SNR), contrast-to-noise ratio, edge sharpness, and equivalent HU of identical region-of-interest (ROI) in congruent image material (15–20). However, studies in veterinary medicine are still lacking.

By comparing quantitative and qualitative image analysis of parallel calculated TUE and VNC image data in dogs without abdominal pathology, this study was performed to verify our hypothesis that the calculated VNC series is comparable to the TUE. By replacing TUE images with VNC images, only one CECT scan would be required, reducing examination and anesthesia time and halving the patient’s radiation dose compared to the commonly applied scan protocol with a native and a post-contrast scan.

## 2 Materials and methods

This retrospective study is based on a total of 283 CT examinations of the abdomen of dogs using SDCT (Philips IQon Spectral CT, Philips Healthcare Germany), which were performed between December 2021 and June 2022 at the Clinic for Small Animals of the University of Veterinary Medicine Hannover. Dogs without radiologically detectable intra-abdominal abnormalities of the organs and major vessels were included in the study. Additionally, we included the available image data of 30 healthy Beagle dogs, which were used in an independent study at the Clinic for Small Animals in September 2022. Dogs with vascular anomalies (i.e., portosystemic shunt) and with CT images visualized lesions of abdominal organs were excluded.

### 2.1 CT protocol

All examinations consisted of a TUE series followed by at least one late venous phase or a combination of arterial-, early (portal-), and late venous phases with the patient in head-first prone recumbency. SDCT scans were acquired with 120–140 kV maximum tube potential, a pitch of 0.6 with a gantry rotation time of 0.5 s, a slice thickness of 2 mm, and a 512 image matrix. The use of automated dose control resulted in a different tube current matched to the size of the patient. CT scans were performed with standardized protocols for the abdomen, using soft tissue and bone kernels with the appropriate bone window (window length: 800; window width: 2,000) and soft tissue window (window length: 60; window width: 350). Image acquisition of the later analyzed CECT venous phase started with a 60-s delay after positive feedback from bolus tracking software with its ROI set in the thoracic aorta, or, if bolus tracking failed, the scan started manually 60–70 s after the initiation of contrast media application. Each patient received 700 mg iobitridol/kgBW (2 mL/kg of Xenetix 350, Guerbet GmbH, Sulzbach, Germany) intravenously administered via a power dual injector system (MEDRAD Stellant/Bayer HealthCare, Leverkusen, Germany).

### 2.2 Quantitative image analysis

The analysis of the image data was performed as a comparative, linked display of the conventional TUE series, conventional post-contrast, VNC series, and monoenergetic reconstruction at 70 keV of the abdomen with system-associated software certified for medical image analysis (IntelliSpace Portal Version 11.x/Philips Healthcare) on a workstation including a monitor certified for image analysis.

First, the TUE images were paired with auto-registration post-processing software for the three reconstructions calculated from post-contrast data: conventional, VNC, and MonoE 70 keV (equivalent to conventional CT). In a few cases, where the auto-registration process failed due to the breathing and movement of the patient, a manual adjustment in the pairing of the images along the x-axis was performed by a *board-certified veterinary* radiologist (KM) and a radiologist in training (PL).

**Figure 1:**
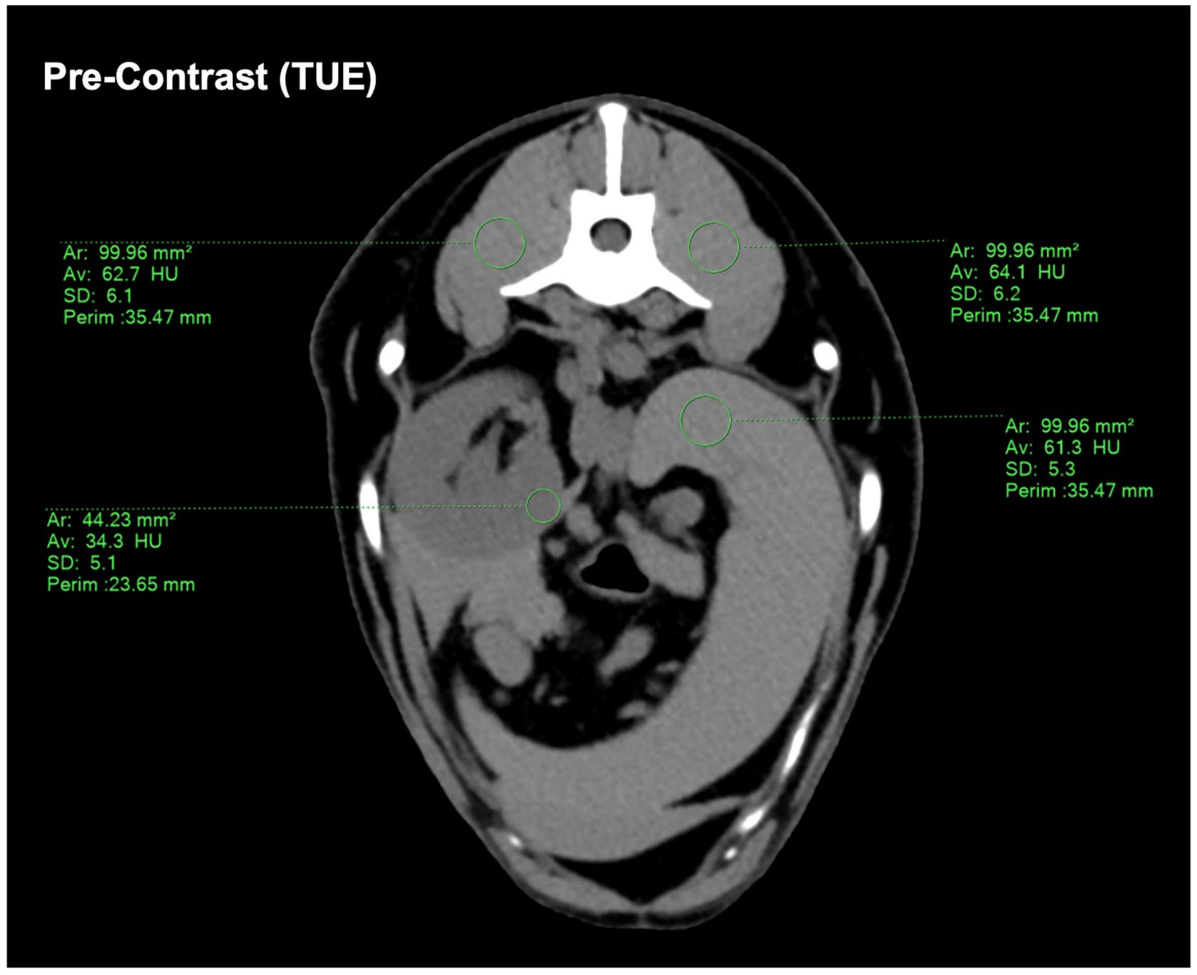
Demonstrating the ROI placement on the conventional pre-contrast CT image. If a size of 1 cm^2^ ± 0.05 cm^2^ was not possible, a ROI size “as large as possible” was chosen. The drawn ROIs were then copy-pasted onto the SBI-data post-contrast images to ensure consistency.

Both examinators assigned multiple, circular regions of interest (ROI) to the TUE images in consensus and transferred them to the paired post-contrast images through copy/paste, therefore being equal in size and location. If practically possible, a uniform size of 1cm^2^ ± 0.05 cm^2^ for individual ROI in TUE was chosen, whereas in structures of smaller size (e.g., pancreas and renal cortex), the largest circular ROI possible was inserted (Figure 1). ROIs were placed in the following organs:

- Liver (1 ROI each, avoiding larger vessels: right lateral hepatic lobe, left lateral hepatic lobe, and caudate lobe).
- Spleen (1 ROI each: splenic head, splenic body, and splenic tail).
- Pancreatic body (1ROI).
- Renal cortex bilaterally (1 ROI each).
- Abdominal aorta (1 ROI) at the level of the 13th thoracic vertebra.
- Paravertebral muscle bilaterally (1 ROI each) at the level of the 13th thoracic vertebra.
- Subcutaneous fat (1 ROI).

The HU difference was determined for each paired ROI in VNC and TUE and classified into four categories based on human medical studies addressing VNC in DECT (15–17, 20):


(1) $${VNC}_{HU}-{TUE}_{HU}=\;\leq 5HU$$

(2) $${VNC}_{HU}-{TUE}_{HU}=\;\leq 10HU$$

(3) $${VNC}_{HU}-{TUE}_{HU}=\;\leq 15HU$$

(4) $${VNC}_{HU}-{TUE}_{HU}=\;>15HU$$

A typical CT window used to evaluate abdominal structures has a window level of 50 HU and a window width of approximately 400 HU (range: −150 HU to +250 HU). This results in a difference of 398 HU, which is equivalent to 398 different shades of gray between black (HU: −150) and white (HU: +250). Ideally, TUE and VNC images should show no difference in HU (±0 HU). The human eye is capable of detecting a grayscale change of up to 6%, which would effectively be 24 shades of gray in an abdominal CT window (21). Following human medical studies such as Sauter et al. (15) and Ananthakrishnan et al. (16), which also dealt with VNC images, even stricter limits were set to be able to classify deviations of HU between complementary TUE and VNC images: a difference in ROIs of ≤10HU between VNC and TUE could be considered “negligible,” while a difference of ≤15HU was still considered “acceptable”. A difference of less than ≤5HU was included in the evaluation to draw attention to the potential of VNC.

For each ROI in TUE and post-contrast derived data, its standard deviation (SD) and attenuation value (HU) were documented, and the signal-to-noise ratio (SNR) of each ROI was assessed by dividing its HU with the corresponding SD.

### 2.3 Qualitative image analysis

Qualitative analysis was performed by two *board-certified veterinary* radiologists (KM, AW-L) in consensus. SDCT datasets and conventional CT images were not blinded, as the image series has been proven to be easily recognized even by radiologists without any experience in using SDCT in a previous study (18).

The overall appearance of the hepatic, splenic, and pancreatic parenchyma as well as the gastrointestinal tract, kidneys, adrenal glands, paraspinal muscles, and major vessels (aorta, vena cava caudalis, and portal vessels) in TUE and VNC images, as well as the efficiency of iodine subtraction in VNC images, were considered for the analysis of the image quality.

A 5-point Likert scale (Table 1) was developed for the qualitative assessment of image noise, comparing SBI and conventional CT, while a modified 5-point Likert scale (Table 2) was developed for the qualitative assessment of iodine subtraction, comparing VNC and TUE.

**Table 1:** Five-point Likert-scale for assessing image noise and image quality in spectral-based images (SBI) compared with images derived by conventional CT.

Image noise and image quality: SBI vs. conventional CT data	
1	SBI markedly worse than conventional CT data
2	SBI mildly worse than conventional CT data
3	SBI equivalent to conventional CT data
4	SBI mildly better than conventional CT data
5	SBI markedly better than conventional CT data

**Table 2:** Modified 5-point Likert scale for assessment of iodine subtraction from VNC images, compared with TUE.

Parenchymal iodine subtraction in VNC	
1	Insufficient subtraction of CM
2	Partly sufficient removal of CM with larger, incomplete areas in the parenchyma
3	Moderate removal of CM wfith incomplete areas in parts of parenchyma
4	Almost complete removal of CM
5	Complete removal of CM

For all structures (except large abdominal vessels), additional assessment was made whether smaller vessels remained recognizable after subtraction of the contrast media.

### 2.4 Statistical methods

Dedicated software was used for statistical data evaluation (GraphPad Prism 9 for macOS Monterey, Version 9.4.1; Microsoft^®^ Excel for Mac, Version 16.65). We grouped and compared VNC with TUE images of each organ/tissue by subtracting the HU of every ROI in VNC from the corresponding ROI in the TUE images. These groups showed a Gaussian distribution using the D’Agostino–Pearson omnibus test. Bland–Altman plots and box and whiskers plots were drawn to graphically evaluate the agreement between VNC and TUE. To test the VNC series and TUE series for equivalence, a two one-sided *t*-test (TOST) was additionally performed using an opposing null hypothesis. In the null hypothesis for the TOST, not the equivalence, but the difference between VNC and TUE images is expected by applying a deviation limit of ≤5, ≤10, or ≤15 HU between both series. If this hypothesis is significantly rejected with a value of *p* < 0.05, an equivalence for both techniques in the specific deviation limit has to be assumed, confirming the original hypothesis of this study (22).

## 3 Results

### 3.1 Study population

Of the total 313 CT examinations (283 client-owned dogs +30 clinic-owned beagles) retrospectively reviewed, the abdomens of only 45 dogs were classified as “radiologically unremarkable” in pre- and post-contrast sequences and thus were enrolled in the study. As the image data of one dog lacked a pre-contrast phase as a comparison, it had to be excluded. Forty-four dogs of the following breeds met our inclusion criteria: beagles (23), mixed breeds (5), French bulldog (2), labrador retriever (2), Australian shepherd (1), Bordeaux mastiff (1), chihuahua (1), miniature American shepherd, old English bulldog (1), miniature dachshund (1), and standard poodle (1). The patients underwent CT examinations for the staging of primary neoplasia (7), for the staging of trauma and the means of planned surgical intervention unrelated to the abdomen (4), for screening for major vascular abnormalities (2), and for orthopedic evaluation (2).

The patients’ mean age was 3 years and 6 months, ranging from 11 months to 10 years and 8 months; 23 dogs were female (4 of them spayed) and 21 were male (2 of them neutered). The mean weight of the patients was 11.8 kg ± 9.4 kg, with a range from 3.4 kg to 43 kg.

### 3.2 Quantitative analysis

A total of 572 ROIs were drawn and copied to the four series for quantitative analysis. Descriptive statistics of the attenuation values in TUE and VNC images are presented in Supplementary Table S1. The difference between TUE and VNC attenuation values was ≤5 HU in 50.87%, ≤10 HU in 78.67%, and ≤15 HU in 91.61% of all compared ROIs. The organ-specific results are detailed in Table 3.

**Table 3:** Number (percentage) of data points, divided by their location, with a difference in TUE and VNC attenuation values ≤5, ≤10, or ≤15.

Region of interest (ROI)	≤5		≤10		≤15	
**Liver** (mean out of three locations)	27/44	61.36%	44/44	100%	44/44	100%
Left lateral lobe	24/44	54.55%	41/44	93.18%	44/44	100%
Right lateral lobe	19/44	43.18%	40/44	90.91%	44/44	100%
Caudate lobe	36/44	81.82%	44/44	100%	44/44	100%
**Spleen** (mean out of three locations)	24/44	54.55%	44/44	100%	44/44	100%
Head	13/44	29.55%	40/44	90.91%	44/44	100%
Body	31/44	70.45%	44/44	100%	44/44	100%
Tail	34/44	77.27%	44/44	100%	44/44	100%
**Pancreas**	34/44	77.27%	41/44	93.18%	44/44	100%
**Muscle** (mean out of left and right)	41/44	93.18%	44/44	100%	44/44	100%
Left	40/44	90.91%	44/44	100%	44/44	100%
Right	40/44	90.91%	44/44	100%	44/44	100%
**Subcutaneous fat**	6/44	13.64%	22/44	50%	39/44	80%
**Renal cortex** (mean out of left and right)	4/44	9.1%	12/44	27.27%	35/44	79.55%
Left	4/44	9.1%	19/44	43.18%	36/44	81.82%
Right	6/44	13.64%	12/44	27.27%	29/44	65.91%
**Aorta**	4/44	9.1%	16/44	36.36%	25/44	56.82%

**Figure 2:**
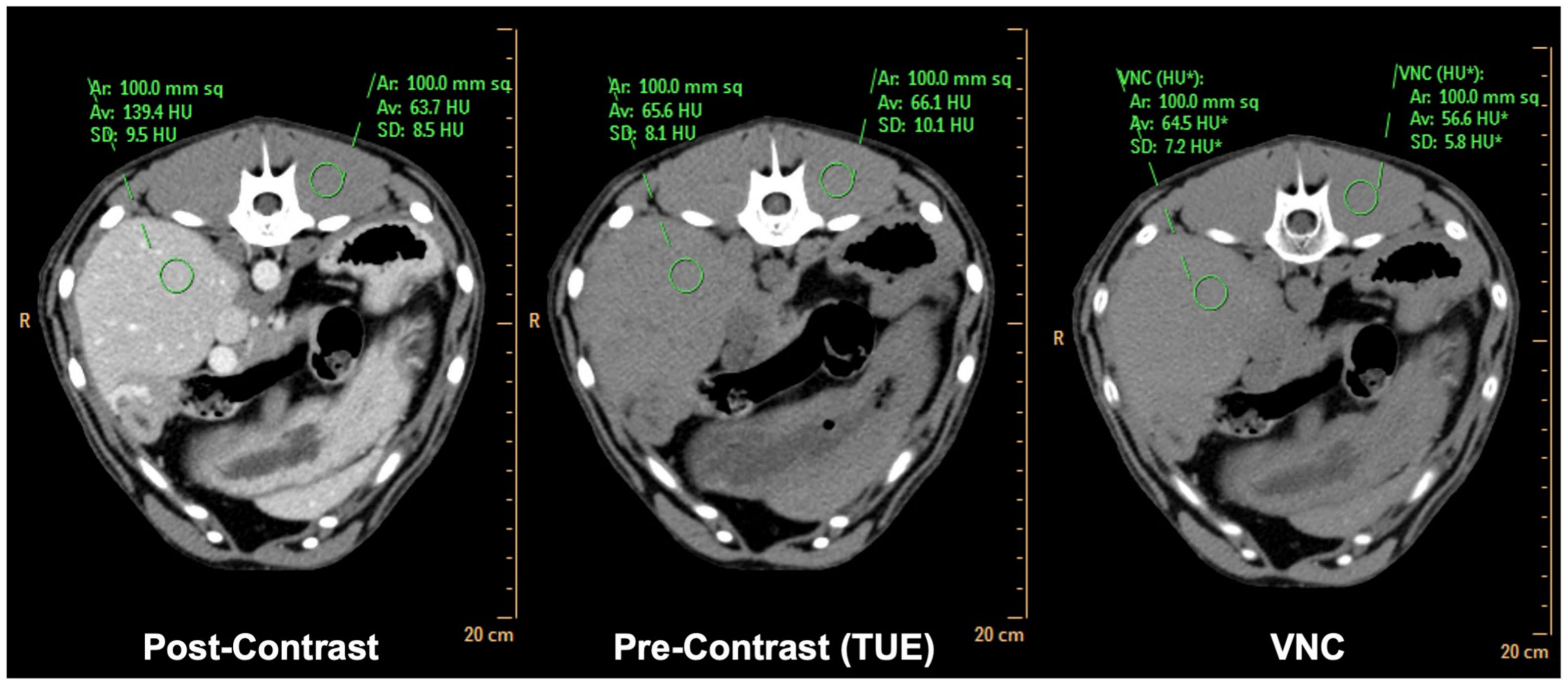
Side-by-side comparison of matched conventional TUE images, conventional post-contrast images and SBI derived VNC images. Note the difference in attenuation values of <2HU in liver and <10HU in paravertebral musculature comparing TUE and VNC.

Bland–Altman plots showed a very good agreement between the two modalities, with the vast majority of the values included within the two error bars (bias ±1.96 × standard deviation), as illustrated for every examined sample in Supplementary Figure S1. An example of these results is illustrated by a side-by-side comparison of conventional and SBI, as shown in Figure 2.

To support these results, TOSTs were performed for every examined organ, with the purpose of proving equivalence between TUE and VNC regarding the ≤5 HU, the ≤10 HU, and the ≤15 HU limits. Considering the ≤10HU limit, equivalence was reached for the liver, spleen, pancreas, and musculature. The TOST results are listed in Table 4, where a *p*-value of < 0.05 implies equivalence of both modalities.

**Table 4:** Test for equivalence using a two one-sided *t*-test (TOST).

Region of interest (ROI)	≤5	≤10	≤15
Liver (mean out of three locations)	0.0026	<0.0001	<0.0001
Spleen (mean out of three locations)	0.9855	<0.0001	<0.0001
Pancreas	<0.0001	<0.0001	<0.0001
Muscle (mean out of left and right)	<0.0001	<0.0001	<0.0001
Subcutaneous fat	1.0	0.1770	<0.0001
Renal cortex (mean out of left and right)	1.0	0.9729	<0.0001
Aorta	1.0	0.9981	0.0608

The tolerance limit (HU) and the derived *p*-values are given for each location.

A *p*-value of < 0.05 indicates that the VNC attenuation values are equivalent. Equivalence was defined with the tolerance limit of ≤5, ≤10, or ≤15 HU difference of VNC toward TUE.

**Figure 3:**
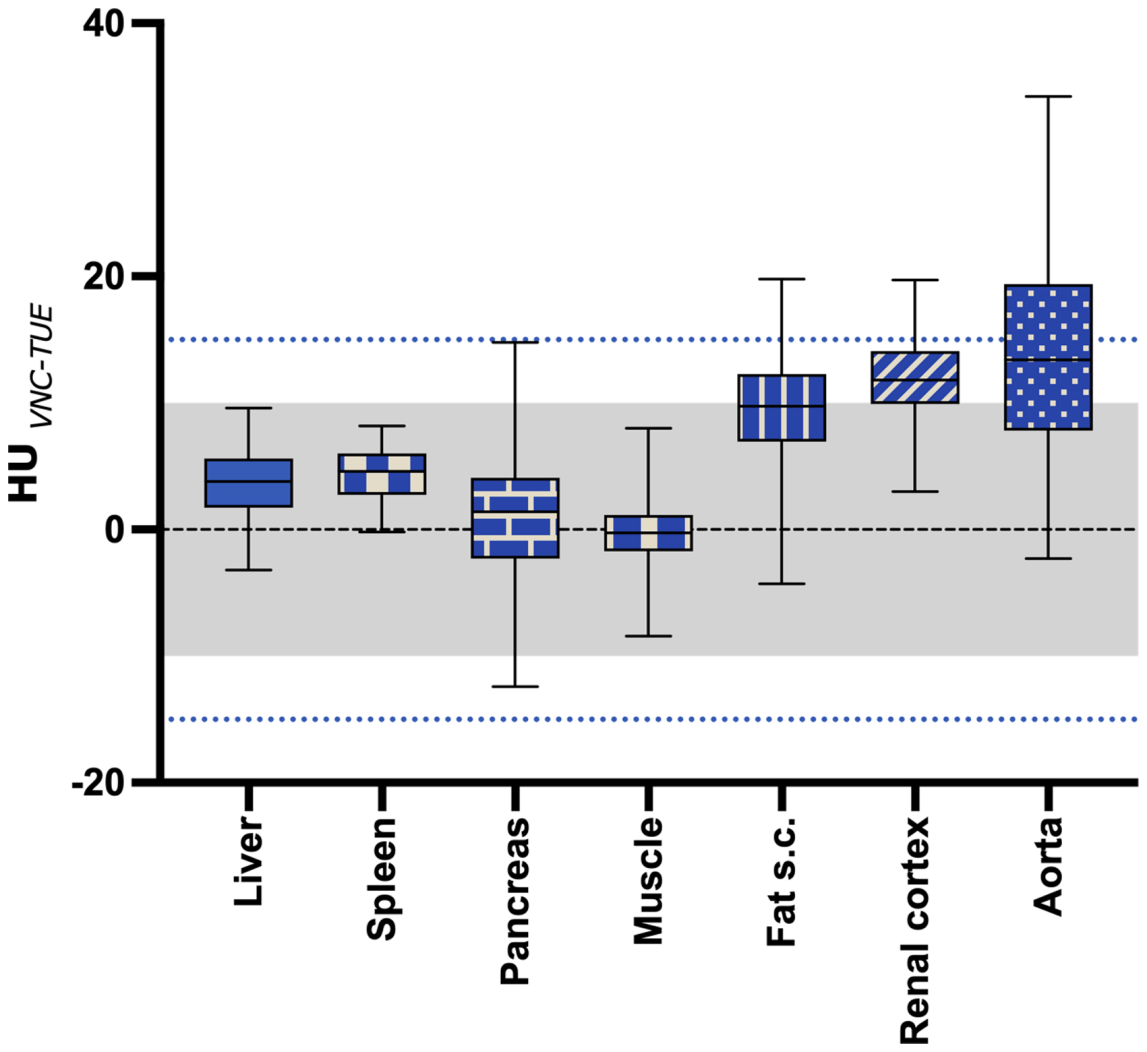
The mean difference between VNC and TUE for all regions of interest. The “negligible” limit of 10HU difference is indicated with the gray shaded area, while the “acceptable” limit of 15HU difference is indicated with the blue dotted line. The lower and upper margins of each box indicate the 25th and 75th percentile. Median is marked by a line.

The mean difference between TUE and VNC was ≤10 HU in the majority of tested regions in the venous phase and is shown with the range of the SD summarized for all regions in Figure 3. Detailed box and whiskers plots for every examined region demonstrating the efficiency of VNC and its comparability with TUE are presented in Supplementary Figure S2, with the CECT as a reference. In summary, the HU of VNC images was in general higher than the ones in the TUE series, especially for ROIs placed in the subcutaneous fat.

The signal-to-noise ratio of SDCT images and conventional CT images was calculated by dividing the specific HU with its standard deviation for each ROI.

### 3.3 Qualitative analysis

The SDCT images in the transverse plane were reviewed in consensus by two board-certified veterinary radiologists (KM and AW-L) regarding image quality and iodine subtraction compared to conventional CT images.

In terms of image quality, SDCT achieved a mean score of 3.27 on a 5-point Likert scale. This tendency for SDCT to have comparable to better image quality than conventional CT is consistent with studies in human medicine (15, 17).

As the second step, the iodine subtraction in the VNC images was evaluated in consensus for the parenchyma of all abdominal organs and major abdominal vessels.

**Figure 4:**
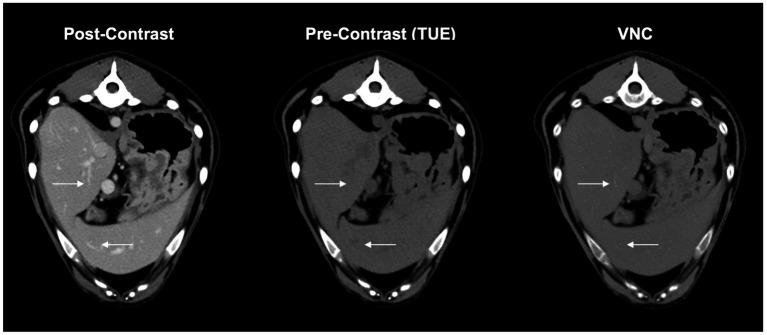
These complementary images in VNC, conventional post contrast and true unenhanced describe the issue of insufficient iodine subtraction in liver and spleen. Minor vessels (arrows) are still visible in VNC-images without affecting the overall diagnostic value of the image itself.

As the VNC algorithm performed insufficiently in subtracting contrast media from small intraparenchymal vessels in all patients, as demonstrated in Figure 4, a modified 5-point Likert scale was used, excluding these vessels from scoring.

The subjective assessment of iodine subtraction in VNC images achieved an average score of at least 4 points or higher on the Likert scale in the aorta, pancreas, portal vein, spleen, liver, and adrenals, while the kidneys, caudal vena cava, and gastrointestinal tract still achieved at least 3.5 points.

All results of the subjective evaluation regarding iodine subtraction and image quality of SBI data vs. conventional CT data are illustrated in Supplementary Table S2.

## 4 Discussion

To the best of our knowledge, this is the first study in veterinary medicine investigating the usability of VNC images derived from spectral detector CT in dogs. In 78.67% of all ROIs compared by VNC with TUE, the difference was ≤10HU. VNC images prove their efficacy in the subtraction of contrast media by showing equivalence to TUE images in the liver, spleen, pancreas, and musculature when applying a limit of ≤10 HU difference in attenuation values. These results are similar to those of a study on people (15, 16).

In 78.67% of all compared ROIs, attenuation values between VNC and TUE images were ≤10 HU, similar to the results in human medicine of Sauter et al. (15) and Jamali et al. (17) using the identical type of SDCT scanner. These promising results increase by up to 91.61% if a limit of 15 HU is considered. Iodine subtraction of VNC images was even more effective than suggested in the study of Ananthakrishnan et al. (16).

The mean difference in attenuation values between VNC and TUE was below 10 HU in every tissue except the renal cortex and aorta and was even below 5 HU in the spleen, liver, pancreas, and paraspinal muscles (Supplementary Table S1). The measured HUs were in general overestimated in VNC compared to TUE images in every tissue except the paraspinal musculature. Interestingly, subcutaneous fat showed the highest deviation of HU in VNC compared to TUE images, even though contrast media uptake of fat is not proven (24). The computation of VNC images is based on water and iodine calculations, which rely on the photoelectric effect and Compton scatter. *Subcutaneous fat is in difference to the parenchymal organs not located on the water ine as reference, but below it*. This could explain the overestimation of HU numbers concerning subcutaneous fat and confirm the correct data sampling (10).

Regarding subcutaneous fat, the comparatively poor performance of the VNC series might not be of clinical relevance as attenuation values still remain clearly negative. In terms of lesions containing increased amounts of intracytoplasmic lipids, such as lipid-rich adenomas, overestimation of VNC might be of clinical relevance, as a threshold of 10 HU for the identification of adrenal adenoma is applied in human medicine by the “European Society of Endocrinology Clinical Practice Guidelines on the management of adrenocortical carcinoma in adults, in collaboration with the European Network for the Study of Adrenal Tumors” (25–27). Such thresholds do not yet exist in veterinary medicine but might have to be considered in future studies when evaluating oncology patients.

While we observed only minor differences in attenuation values between TUE and VNC images of the musculature and the parenchyma of the liver and spleen, with 100% of the mean attenuation value differences below 10 HU, we still experienced a distinct lack of subtracting iodine from small intraparenchymal vessels, similar to Laukamp et al. (20). There is evidence that the VNC algorithm works only to a limited extent when there is a high concentration of iodine-based contrast medium in narrowly defined areas. This could be explained by the partial volume effect in CT image reconstruction. Once a voxel is composed of different materials, the final HU in the cross-sectional image can only result from an average estimate of the individual attenuation values of the materials involved. Because the differences in these attenuation values at the boundary between a contrast-enhancing vessel and the surrounding parenchyma are very large, the HU will be an average estimate that lies between the very high HU from iodine in the lumen (approximately 100–500 HU) and the low HU from surrounding tissue (approximately 35–40 HU). This estimated value does not reflect the attenuation value of iodine in the voxel, which could then also cause the iodine subtraction of the VNC image to be insufficient. Reduced efficiency in removing iodine from small vessels was most obvious in the liver, less so in the spleen, and almost non-existent in the pancreas and adrenal glands.

This could further explain the poorer iodine subtraction in the renal cortex, as this region is rich in multiple very small vessels (arcuate and interlobular arteries and veins). This poorer correlation of TUE with VNC images in the renal cortices leads to a clear distinction between the cortex and medulla in VNC images compared to the TUE series.

The partial volume effect also explains why iodine subtraction in large vessels (aorta and caudal vena cava) achieves better values in the subjective assessment because the partial volume effect does not have such a significant impact. Incomplete subtraction of contrast medium was only present in large vessels where the contrast medium was incompletely mixed with blood. In our study, we found this effect mainly in the ventral aspect of the caudal vena cava and bifurcations of the portal vein, focusing on the evaluation of the venous phase, which has been similarly described in a publication from human medicine due to beam-hardening artifacts by non-mixed contrast material in major vessels (28). The same effect could potentially occur in the aorta when evaluating VNC images calculated from the arterial phase of a post-contrast scan, but this has not been further assessed in our study.

We also included the pancreas as a parenchymal organ in our study. With a mean difference in VNC to TUE attenuation values of 1.44 (CI 95% −0.051 to 2.937), the iodine subtraction performed very well in this organ, only working more precisely in the paraspinal musculature by reaching a mean difference of −0.36 (CI 95% −1.208 to 0.481). It seems reasonable that the performance of iodine subtraction depends on distribution pattern, accumulation, concentration of iodine in the respective tissue, and interindividual variability, as shown in human medicine (24). The study by Zopfs et al. (24) supplies detailed information on contrast media distribution in abdominal organs and tissues, based on the assessment of average iodine concentration and iodine perfusion ratio. Both parameters demonstrated similar values for the liver, spleen, and pancreas in humans; therefore, the effectiveness of the iodine subtraction algorithm seems comprehensible in this species. No such values exist for our canine patients and would need to be proven. Interestingly, Zopf et al. showed the highest iodine concentration and perfusion rate in the renal cortex, even slightly exceeding the values of major vessels such as the aorta and portal vein. Both renal cortex and abdominal aorta showed nearly identical results in the mean difference of attenuation values between TUE and VNC in our analysis, further supporting a correlation between organ-specific contrast medium distribution and the efficacy of iodine subtraction.

The entire gastrointestinal tract (GIT) was also included in the subjective analysis. In VNC images, a clear separation of wall layering in the GIT was still obvious compared to the TUE images. Future studies need to address the question, whether this makes a difference in the interpretation of gastrointestinal pathologies.

In order to include patients in our study population, all CT scans of the abdomen performed on dogs and cats between December 2021 and October 2022 were reviewed. Patients were excluded from the study population when they did not fulfill the criterion of normal due to small mineralizations associated with the kidneys that were visible in the native scan. We noticed that these mineralizations were sometimes only visible in the TUE images, while they were absent in the VNC image. This is especially important as such small mineralizations might be missed in post-contrast scans due to the accumulation of contrast medium in the diverticuli and renal pelvis. This problem has been pointed out in various studies in human medicine, especially when structures smaller than 3 mm were involved (23). Although this problem is not part of the current study as these patients have been excluded, it highlights an important problem of iodine subtraction in clinical patients. Future studies are needed to evaluate the usefulness of VNC images for clinical patients. In human patients, VNC image data are used in combination with other SBI algorithms, such as iodine concentration and z-effective number maps, to minimize such VNC errors (29).

The image quality of SDCT-derived images was equal or superior in every subjective evaluation and proved to have a better SNR compared to conventional CT images in statistical analysis. These results were expected, as effective image noise reduction algorithms were implemented early in the development of DECT (30, 31). Additionally, due to the simultaneous detection of low- and high-energy signals, it is possible to suppress beam-hardening artifacts (9). With SDCT, it is also possible to reduce certain types of artifacts, for example, streaking artifacts due to metal implants, by choosing a certain level of monoenergetic image. This feature was not used in our study but would additionally improve image quality for clinical patients.

The present study has several limitations. Our study population was limited and not large enough to draw conclusions about the influence of breed, age, sex, and body condition on the VNC images. As dogs have a huge variation in body size, shape, and weight, one needs to be cautious in applying our results to the overall canine population, especially as we did not include the body condition score in our assessment. Another major limitation is that we only included CT scans of patients with a radiologically normal appearance of the abdominal organs. Future studies will need to investigate whether VNC images can be used for clinical patients with various pathologies. Finally, our study applied the iodine subtraction algorithm only to SDCT images acquired in the venous phase. Other contrast phases might differ in their VNC assessment, for example, and as aforementioned, the contrast medium did not completely dissolve in the blood in larger vessels or specific organs.

## 5 Conclusion

In conclusion, spectral CT-derived VNC images showed no significant deviation from TUE images in over 78% of all ROIs by applying a limit of ±10 HU difference as negligible. The iodine subtraction algorithm performed most accurately in the liver, spleen, pancreas, and paraspinal musculature. If VNC images should substitute for TUE images in clinical patients, future studies need to first address the behavior of VNC for the various abdominal pathologies. The use of virtual subtraction of contrast medium in clinical patients reduces CT examination and anesthesia time and reduces the patient’s exposure to radiation.

## Data availability statement

The raw data supporting the conclusions of this article will be made available by the authors, without undue reservation.

## Ethics statement

The animal study was approved by LAVES (Niedersächsisches Landesamt für Verbraucherschutz und Lebensmittelsicherheit), also due to its retrospective character. The study was conducted in accordance with the local legislation and institutional requirements. A direct, written consent of the animal owners for the use of the patient data in this certain study was not obtained, as the anonymized use of the data in general is approved by signature during the admission of the patients in the registration certificate.

## Author contributions

PL: literature research, data and image acquisition, planning and conducting the study, calculating and interpreting results, writing and submitting article, involved in every study process. MB: literature research, planning and conducting the study, reviewing and edditing article, involved in every study process. AW-L: subjective and objective image analysis, review of article, and study planning. HV: supervisor, review of article, and study planning. SM: review and planning of the statistical analysis. KM: senior and last author, main supervisor of the complete study and involved in every study process, image analysis (subjective and objective), and final review of article. All authors contributed to the article and approved the submitted version.
