# Supplementary material for: Virtual non-contrast images of detector-based spectral computed tomography in dogs: a promising alternative to true non-contrast images in veterinary medicine
Source: Front Vet Sci. 2023 Dec 1;10:1251535. doi: 10.3389/fvets.2023.1251535 (PMC10722308; doi:10.3389/fvets.2023.1251535)
Supplement: Supplementary file 1 [file Table_1.DOCX]

| Region of Interest (**ROI**) |  | **TUE** Mean,  SD |  | **TUE** Min |  | **TUE** Max |  | **TUE**  Difference |  | **VNC** Mean, SD |  | **VNC** Min |  | **VNC** Max |  | **VNC** Difference |  | **VNC - TUE**  Mean Difference |  | **VNC - TUE**  CI 95% |  | **VNC - TUE**  *p*-value |
| --- | --- | --- | --- | --- | --- | --- | --- | --- | --- | --- | --- | --- | --- | --- | --- | --- | --- | --- | --- | --- | --- | --- |
| **Liver**  (Mean out of 3 locations) |  | 59.08 ± 3.98 |  | 49.73 |  | 67.27 |  | 17.54 |  | 62.88 ± 4.6 |  | 50.03 |  | 71.67 |  | 21.64 |  | 3.79 |  | 2.902 to 4.683 |  | <0.0001 |
| **Spleen**  (Mean out of 3 locations) |  | 64.25 ± 4.33 |  | 42.67 |  | 72.1 |  | 29.43 |  | 68.66 ± 5.16 |  | 42.5 |  | 75.57 |  | 33.07 |  | 4.42 |  | 3.813 to 5.018 |  | <0.0001 |
| **Pancreas** |  | 54.20 ± 4.53 |  | 43.7 |  | 61.5 |  | 17.8 |  | 55.64 ± 5.14 |  | 41.2 |  | 64.2 |  | 23 |  | 1.44 |  | -0.051 to 2.937 |  | 0.0580 |
| **Muscle**  (Mean out of left and right) |  | 59.3 ± 4.20 |  | 41.8 |  | 64.35 |  | 22.55 |  | 58.93 ± 3.65 |  | 44 |  | 63.05 |  | 19.05 |  | - 0.3636 |  | -1.208 to 0.481 |  | 0.3898 |
| **Subcutaneous** **Fat** |  | -106.7 ± 15.23 |  | -129.9 |  | -66.2 |  | 63.7 |  | -97.15 ± 13.32 |  | -119.2 |  | -69.4 |  | 49.8 |  | 9.539 |  | 8.122 to 10.96 |  | <0.0001 |
| **Renal cortex**  (Mean out of left and right) |  | 37.53 ± 4.19 |  | 26.55 |  | 45.65 |  | 19.1 |  | 49.21 ± 5.79 |  | 31 |  | 58.3 |  | 27.3 |  | 11.68 |  | 10.47 to 12.88 |  | <0.0001 |
| **Aorta** |  | 37.38 ± 5.41 |  | 19.5 |  | 49.8 |  | 29.1 |  | 50.98 ± 8.33 |  | 26.6 |  | 74.1 |  | 47.5 |  | 13.6 |  | 11.46 to 15.74 |  | <0.0001 |

**Supplementary Table 1:** Mean, standard deviation (SD), minimum, maximum, difference, and mean difference in HU, as well as 95% confidence interval and *p*- value for each location

|  | ***Dog*** | | | | | | | | | | | | | | | | | | | | | | | | | | | | |  |  |
| --- | --- | --- | --- | --- | --- | --- | --- | --- | --- | --- | --- | --- | --- | --- | --- | --- | --- | --- | --- | --- | --- | --- | --- | --- | --- | --- | --- | --- | --- | --- | --- |
|  | ***1*** | ***2*** | | ***3*** | | ***4*** | | ***5*** | | ***6*** | | ***7*** | | ***8*** | | ***9*** | | ***10*** | | ***11*** | | ***12*** | | ***13*** | | ***14*** | | ***15*** | |  |  |
|  | ***Bodyweight (kg)*** | | | | | | | | | | | | | | | | | | | | | | | | | | | | |  |  |
|  | **20.4** | **15.8** | | **13.6** | | **19** | | **17** | | **13.6** | | **3.4** | | **21.8** | | **23.6** | | **51** | | **16.5** | | **4** | | **13.8** | | **17.6** | | **43** | |  |  |
| **Location** | ***Score: Iodine Subtraction*** | | | | | | | | | | | | | | | | | | | | | | | | | | | | |  | **Average Score**  **Location** |
| **Liver** | 5 | 4 | | 5 | | 4 | | 4 | | 4 | | 4 | | 5 | | 5 | | 5 | | 5 | | 5 | | 5 | | 5 | | 5 | |  | **4.67** |
| **Spleen** | 5 | 3 | | 5 | | 4 | | 3 | | 4 | | 4 | | 5 | | 5 | | 5 | | 5 | | 5 | | 5 | | 5 | | 5 | |  | **4.53** |
| **Pancreas** | 5 | 4 | | 5 | | 5 | | 5 | | 4 | | 5 | | 5 | | 5 | | 5 | | 5 | | 5 | | 5 | | 5 | | 5 | |  | **4.87** |
| **Kidney** | 5 | 3 | | 3 | | 3 | | 3 | | 3 | | 3 | | 4 | | 4 | | 5 | | 3 | | 4 | | 4 | | 4 | | 4 | |  | **3.67** |
| **Adrenal glands** | 4 | 4 | | 5 | | 5 | | 3 | | 4 | | 5 | | 5 | | 5 | | 5 | | 4 | | 4 | | 5 | | 4 | | 4 | |  | **4.40** |
| **GIT** | 4 | 3 | | 4 | | 4 | | 2 | | 3 | | 3 | | 4 | | 4 | | 4 | | 4 | | 4 | | 4 | | 4 | | 4 | |  | **3.67** |
| **VCC** | 2 | 1 | | 5 | | 5 | | 1 | | 1 | | 5 | | 5 | | 2 | | 5 | | 5 | | 3 | | 5 | | 5 | | 5 | |  | **3.67** |
| **Aorta** | 5 | 5 | | 5 | | 5 | | 5 | | 5 | | 5 | | 5 | | 5 | | 5 | | 5 | | 5 | | 5 | | 5 | | 5 | |  | **5.00** |
| **PV** | 5 | 5 | | 5 | | 5 | | 4 | | 5 | | 5 | | 5 | | 5 | | 5 | | 5 | | 5 | | 5 | | 5 | | 5 | |  | **4.93** |
|  |  |  | |  | |  | |  | |  | |  | |  | |  | |  | |  | |  | |  | |  | |  | |  |  |
| **Average Score**  **Patient** | **4.44** | **3.56** | | **4.67** | | **4.44** | | **3.33** | | **3.67** | | **4.33** | | **4.78** | | **4.44** | | **4.89** | | **4.56** | | **4.44** | | **4.78** | | **4.67** | | **4.67** | |  | **4.38** |
|  |  | | | | | | | | | | | | | | | | | | | | | | | | | | | | |  |  |
|  | ***Score: Image Quality / Image Noise*** | | | | | | | | | | | | | | | | | | | | | | | | | | | | |  | **Average Score**  **Quality** |
|  | **4** | | **4** | | **4** | | **4** | | **4** | | **5** | | **5** | | **4** | | **4** | | **4** | | **4** | | **5** | | **4** | | **4** | | **4** |  | **4.2** |

**Supplementary Table 2:** Iodine subtraction score and Image Quality / Image Noise score for 44 dogs in every location included in the study.

Note, the almost complete iodine subtraction with an average score of at least 4.2 points in VNC images and the overall superior image quality of SBI-images.

|  | ***Dog*** | | | | | | | | | | | | | | | | | | | | | | | | | | | | |  |  |
| --- | --- | --- | --- | --- | --- | --- | --- | --- | --- | --- | --- | --- | --- | --- | --- | --- | --- | --- | --- | --- | --- | --- | --- | --- | --- | --- | --- | --- | --- | --- | --- |
|  | ***16*** | ***17*** | | ***18*** | | ***19*** | | ***20*** | | ***21*** | | ***22*** | | ***23*** | | ***24*** | | ***25*** | | ***26*** | | ***27*** | | ***28*** | | ***29*** | | ***30*** | |  |  |
|  | ***Bodyweight (kg)*** | | | | | | | | | | | | | | | | | | | | | | | | | | | | |  |  |
|  | ***9.4*** | ***9.9*** | | ***8.2*** | | ***8.2*** | | ***8.5*** | | ***8.2*** | | ***6.7*** | | ***7.6*** | | ***6.3*** | | ***7.0*** | | ***8.7*** | | ***8.1*** | | ***9.0*** | | ***9.5*** | | ***6.8*** | |  |  |
| **Location** | ***Score: Iodine Subtraction*** | | | | | | | | | | | | | | | | | | | | | | | | | | | | |  | **Average Score**  **Location** |
| **Liver** | 5 | 3 | | 5 | | 4 | | 4 | | 5 | | 4 | | 4 | | 4 | | 5 | | 4 | | 5 | | 4 | | 4 | | 4 | |  | **4.27** |
| **Spleen** | 5 | 3 | | 5 | | 5 | | 3 | | 4 | | 4 | | 5 | | 5 | | 5 | | 4 | | 5 | | 5 | | 4 | | 5 | |  | **4.47** |
| **Pancreas** | 5 | 4 | | 5 | | 5 | | 5 | | 5 | | 5 | | 5 | | 5 | | 5 | | 5 | | 5 | | 5 | | 5 | | 5 | |  | **4.93** |
| **Kidney** | 4 | 3 | | 3 | | 4 | | 3 | | 4 | | 4 | | 3 | | 4 | | 4 | | 4 | | 4 | | 4 | | 3 | | 4 | |  | **3.67** |
| **Adrenal glands** | 4 | 4 | | 4 | | 5 | | 4 | | 4 | | 4 | | 4 | | 4 | | 4 | | 4 | | 4 | | 4 | | 4 | | 4 | |  | **4.07** |
| **GIT** | 3 | 3 | | 3 | | 4 | | 3 | | 3 | | 3 | | 4 | | 4 | | 4 | | 3 | | 3 | | 4 | | 4 | | 4 | |  | **3.47** |
| **VCC** | 4 | 3 | | 3 | | 4 | | 3 | | 4 | | 4 | | 4 | | 3 | | 4 | | 4 | | 4 | | 4 | | 3 | | 4 | |  | **3.67** |
| **Aorta** | 5 | 5 | | 5 | | 5 | | 5 | | 5 | | 5 | | 5 | | 5 | | 5 | | 5 | | 5 | | 5 | | 5 | | 5 | |  | **5.00** |
| **PV** | 5 | 5 | | 5 | | 5 | | 5 | | 5 | | 5 | | 5 | | 5 | | 5 | | 4 | | 4 | | 5 | | 5 | | 5 | |  | **4.87** |
|  |  |  | |  | |  | |  | |  | |  | |  | |  | |  | |  | |  | |  | |  | |  | |  |  |
| **Average Score**  **Patient** | **4.44** | **3.67** | | **4.22** | | **4.56** | | **3.89** | | **4.33** | | **4.22** | | **4.33** | | **4.33** | | **4.56** | | **4.11** | | **4.33** | | **4.44** | | **4.11** | | **4.44** | |  | **4.27** |
|  |  | | | | | | | | | | | | | | | | | | | | | | | | | | | | |  |  |
|  | ***Score: Image Quality / Image Noise*** | | | | | | | | | | | | | | | | | | | | | | | | | | | | |  | **Average Score**  **Quality** |
|  | **4** | | **4** | | **2** | | **2** | | **3** | | **3** | | **3** | | **3** | | **3** | | **2** | | **2** | | **2** | | **2** | | **2** | | **2** |  | **2.6** |

|  | ***Dog*** | | | | | | | | | | | | | | | | | | | | | | | | | | | | |  |  |
| --- | --- | --- | --- | --- | --- | --- | --- | --- | --- | --- | --- | --- | --- | --- | --- | --- | --- | --- | --- | --- | --- | --- | --- | --- | --- | --- | --- | --- | --- | --- | --- |
|  | ***31*** | ***32*** | | ***33*** | | ***34*** | | ***35*** | | ***36*** | | ***37*** | | ***38*** | | ***39*** | | ***40*** | | ***41*** | | ***42*** | | ***43*** | | ***44*** | |  | |  |  |
|  | ***Bodyweight (kg)*** | | | | | | | | | | | | | | | | | | | | | | | | | | | | |  |  |
|  | ***8.5*** | ***10.8*** | | ***6.8*** | | ***7.2*** | | ***9.6*** | | ***9.9*** | | ***6.8*** | | ***7.3*** | | ***6.3*** | | ***6.7*** | | ***7.9*** | | ***7.7*** | | ***6.5*** | | ***7.3*** | |  | |  |  |
| **Location** | ***Score: Iodine Subtraction*** | | | | | | | | | | | | | | | | | | | | | | | | | | | | |  | **Average Score**  **Location** |
| **Liver** | 4 | 4 | | 4 | | 5 | | 5 | | 4 | | 5 | | 4 | | 5 | | 5 | | 5 | | 4 | | 4 | | 5 | |  | |  | **4.50** |
| **Spleen** | 4 | 4 | | 5 | | 5 | | 5 | | 5 | | 4 | | 4 | | 5 | | 4 | | 5 | | 5 | | 4 | | 5 | |  | |  | **4.57** |
| **Pancreas** | 5 | 5 | | 5 | | 5 | | 5 | | 5 | | 5 | | 5 | | 5 | | 5 | | 5 | | 5 | | 5 | | 5 | |  | |  | **5.00** |
| **Kidney** | 4 | 3 | | 4 | | 4 | | 3 | | 4 | | 4 | | 3 | | 4 | | 4 | | 4 | | 4 | | 4 | | 4 | |  | |  | **3.79** |
| **Adrenal glands** | 3 | 3 | | 3 | | 4 | | 4 | | 4 | | 4 | | 4 | | 4 | | 3 | | 4 | | 4 | | 4 | | 4 | |  | |  | **3.71** |
| **GIT** | 3 | 3 | | 4 | | 4 | | 4 | | 4 | | 4 | | 3 | | 3 | | 4 | | 3 | | 4 | | 3 | | 4 | |  | |  | **3.57** |
| **VCC** | 4 | 3 | | 4 | | 4 | | 3 | | 3 | | 3 | | 3 | | 3 | | 4 | | 4 | | 4 | | 4 | | 3 | |  | |  | **3.50** |
| **Aorta** | 5 | 5 | | 5 | | 5 | | 5 | | 5 | | 5 | | 5 | | 5 | | 5 | | 5 | | 5 | | 5 | | 5 | |  | |  | **5.00** |
| **PV** | 5 | 4 | | 5 | | 5 | | 4 | | 5 | | 5 | | 5 | | 5 | | 5 | | 5 | | 5 | | 5 | | 5 | |  | |  | **4.86** |
|  |  |  | |  | |  | |  | |  | |  | |  | |  | |  | |  | |  | |  | |  | |  | |  |  |
| **Average Score**  **Patient** | **4.11** | **3.78** | | **4.33** | | **4.56** | | **4.22** | | **4.33** | | **4.33** | | **4.00** | | **4.33** | | **4.33** | | **4.44** | | **4.44** | | **4.22** | | **4.44** | |  | |  | **4.28** |
|  |  | | | | | | | | | | | | | | | | | | | | | | | | | | | | |  |  |
|  | ***Score: Image Quality / Image Noise*** | | | | | | | | | | | | | | | | | | | | | | | | | | | | |  | **Average Score**  **Quality** |
|  | **2** | | **2** | | **3** | | **3** | | **3** | | **3** | | **3** | | **3** | | **3** | | **3** | | **4** | | **3** | | **3** | | **4** | |  |  | **3.0** |
