# Supplementary figures and images for: Virtual non-contrast images of detector-based spectral computed tomography in dogs: a promising alternative to true non-contrast images in veterinary medicine
Source: Front Vet Sci. 2023 Dec 1;10:1251535. doi: 10.3389/fvets.2023.1251535 (PMC10722308; doi:10.3389/fvets.2023.1251535)

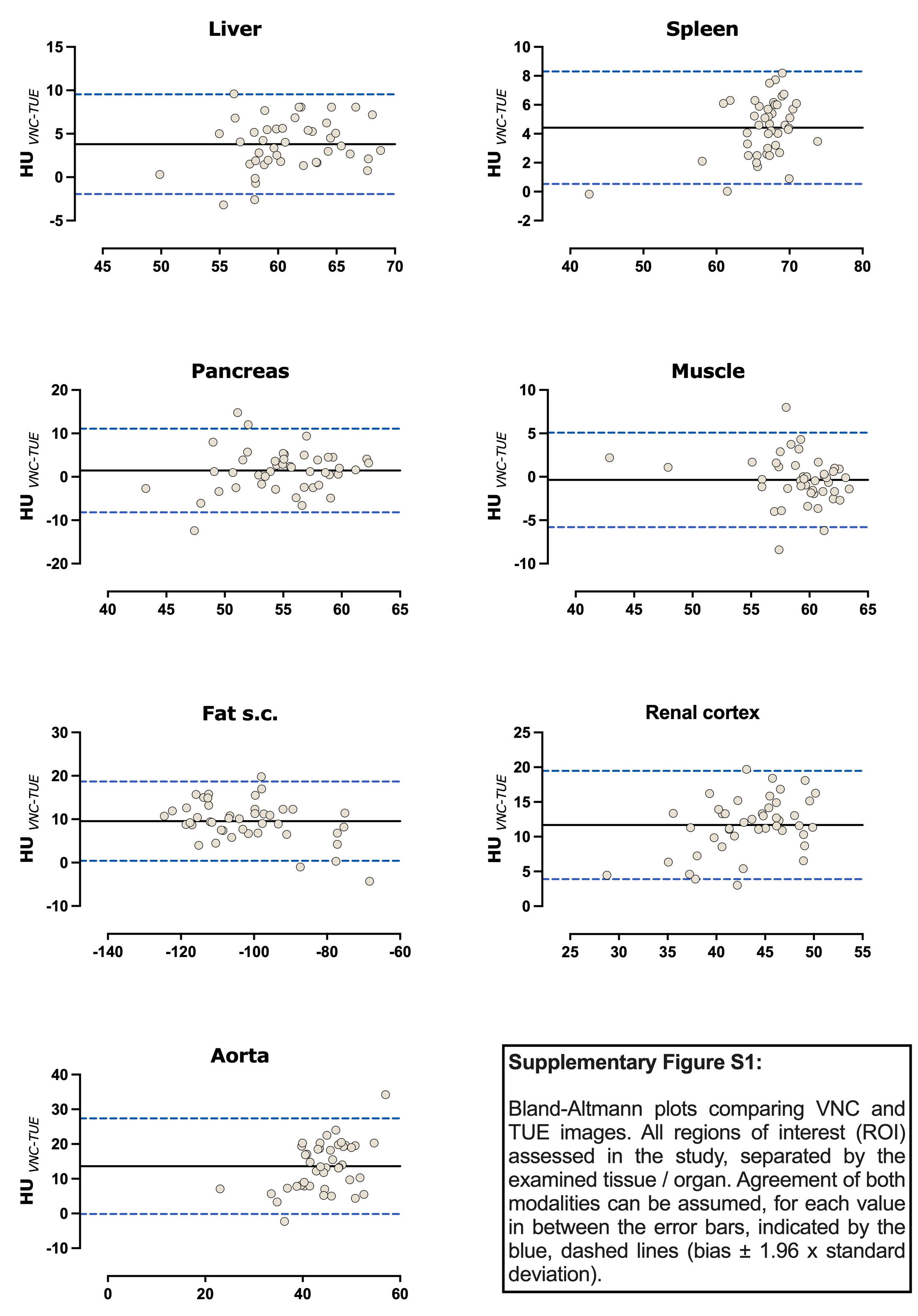

Supplement: Supplementary file 2 [file Image_1.tiff]

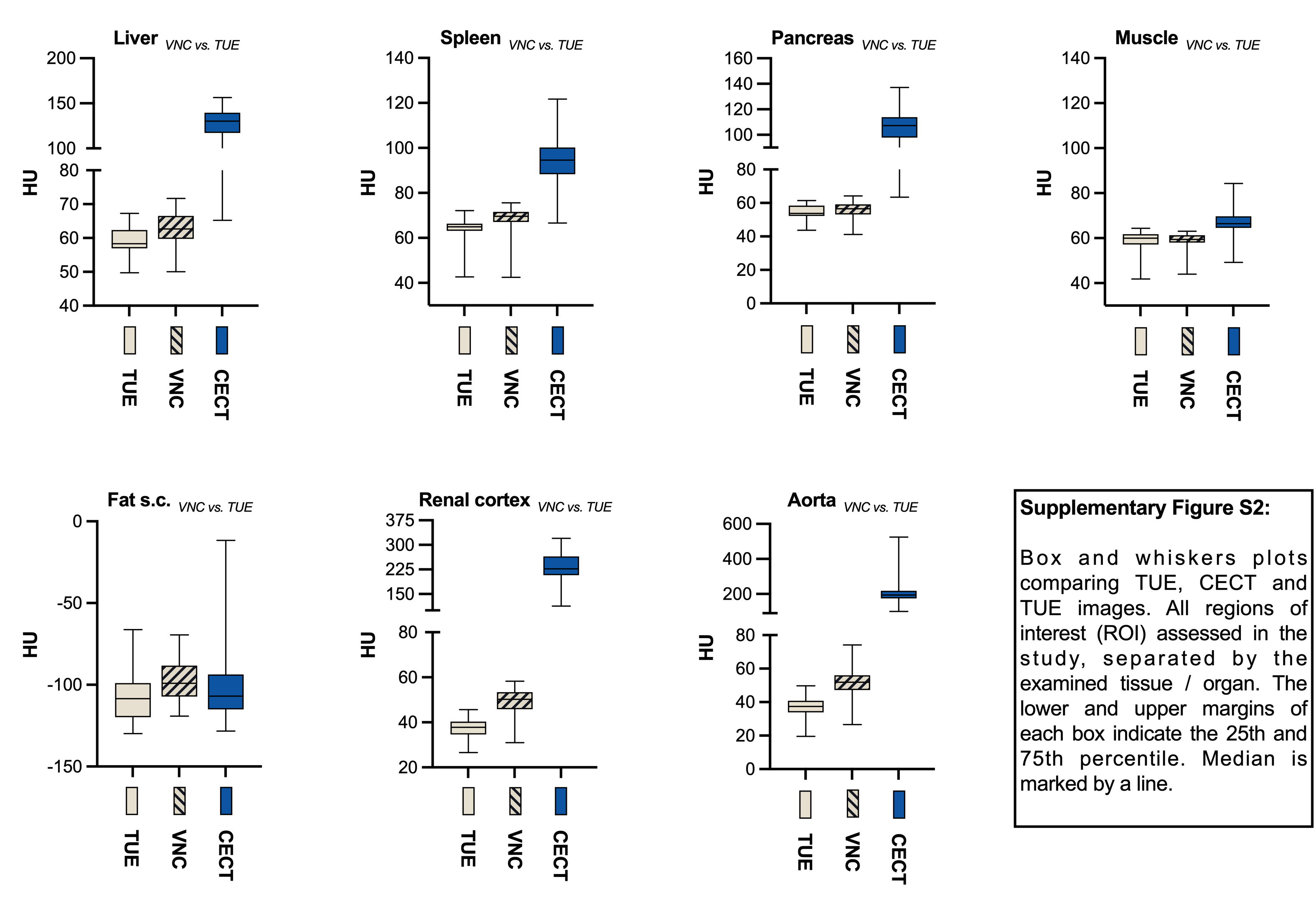

Supplement: Supplementary file 3 [file Image_2.tiff]
